# Induction of Apoptosis by Costunolide in Bladder Cancer Cells is Mediated through ROS Generation and Mitochondrial Dysfunction

**DOI:** 10.3390/molecules18021418

**Published:** 2013-01-24

**Authors:** Azhar Rasul, Rui Bao, Mahadev Malhi, Bing Zhao, Ichiro Tsuji, Jiang Li, Xiaomeng Li

**Affiliations:** 1 Dental Hospital, Jilin University, Changchun 130041, China; 2 The Key Laboratory of Molecular Epigenetics of MOE, Institute of Genetics and Cytology, Northeast Normal University, Changchun 130024, China; 3 Department of Public Health, Tohoku University, Sendai 9808576, Japan

**Keywords:** bladder cancer, T24 cells, costunolide, apoptosis, reactive oxygen species

## Abstract

Despite the availability of several therapeutic options, a safer and more effective modality is urgently needed for treatment of bladder cancer. Costunolide, a member of sesquiterpene lactone family, possesses potent anticancer properties. In this study, for the first time we investigated the effects of costunolide on the cell viability and apoptosis in human bladder cancer T24 cells. Treatment of T24 cells with costunolide resulted in a dose-dependent inhibition of cell viability and induction of apoptosis which was associated with the generation of ROS and disruption of mitochondrial membrane potential (Δψm). These effects were significantly blocked when the cells were pretreated with N-acetyl- cysteine (NAC), a specific ROS inhibitor. Exposure of T24 cells to costunolide was also associated with increased expression of Bax, down-regulation of Bcl-2, survivin and significant activation of caspase-3, and its downstream target PARP*.* These findings provide the rationale for further *in vivo* and clinical investigation of costunolide against human bladder cancer.

## 1. Introduction

Urinary bladder cancer is one of the most common urological malignancie*s* worldwide. More than 12 million new cases of cancer occur annually worldwide. Of those 5.4 million occur in developed countries and 6.7 million in developing countries [1]. In 2012, approximately 37,510 new urinary bladder cancer cases will be diagnosed and 14,880 will die in the United States [2]. In recent years, bladder cancer has been usually cured with surgery, chemotherapy, and combinations of chemotherapy and radiotherapy, but they all have associated limitations [1]. Prevailing treatment options have limited therapeutic success in human bladder cancer. Hence, the current therapy for bladder cancer is not satisfactory and better therapeutic options are immediately required to develop a more effective therapy for bladder cancer that can reduce the recurrence rate, decrease side effects, and increase overall survival.

Over the last decade, many reports revealed that phytochemicals targeting ROS metabolism can selectively kill cancer cells by raising the level of ROS above a toxic threshold. Since cancer cells show higher levels of endogenous ROS compared with their normal cells, the toxic threshold can be easily achieved in cancer cells [3,4]. In the current study, we carried out high throughput screening of compound library from Chinese herbs, using the bladder cancer cell line T24, in the presence or absence of NAC, a specific ROS inhibitor. This screening strategy helped us to identify natural anticancer compounds targeting ROS mediated apoptosis in bladder cancer cells. Costunolide, a natural compound that belongs to the sesquiterpene lactone family, was identified as a potent growth inhibitor of bladder cancer cells during screening. Sesquiterpene lactones, due to their anti-neoplastic and anti-inflammatory activity, have attracted considerable attention in pharmacological research [5,6]. As a medicine, costunolide is a well known sesquiterpene lactone, which is used as popular herbal remedies, with anti-ulcer [7], anti-inflammatory [8], anti-fungal [9,10], anti-viral properties [11], and inhibitory effects against cellular production of melanin [12]. It has also been documented that costunolide is involved to inhibit the expression of inducible nitric oxide synthase [13] and the DNA-binding activity of NF-κB [14]. Moreover, costunolide potentiated 1,25-(OH)2D3-induced differentiation in HL-60 promyelocytic leukemia cells [15,16,17] via interference with NF-κB activation. Further studies demonstrated that costunolide has anti-tumor potential by inhibiting proliferation, inducing apoptosis and reducing invasion and metastasis of a wide variety of tumor cells as we reviewed recently [18]. However, the effects of costunolide on human bladder cancer T24 cells were still unknown. Therefore, the objectives of present study were two-fold; to explore the effects of costunolide on the proliferation of T24 cells and to determine the role ROS in costunolide-induced apoptosis in bladder cancer cells with a therapeutic potential. Results showed that costunolide effectively inhibited the proliferation of T24 cells through inducing the apoptosis, which is mediated through ROS generation, mitochondrial dysfunction and activation of caspase-3 and its downstream target Poly (ADP-ribose) polymerase (PARP).

## 2. Results and Discussion

### 2.1. Costunolide Exerted Anti-Proliferation Activity in T24 Cells

**Figure 1:**
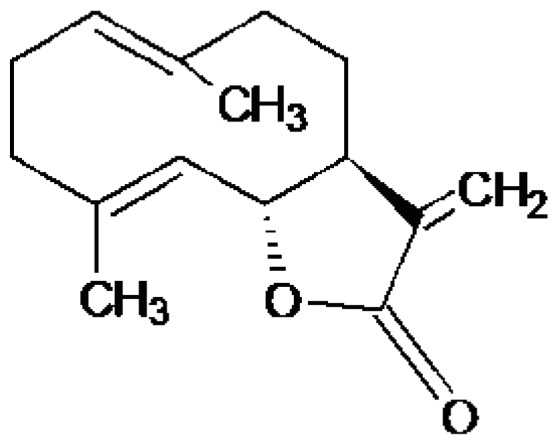
The chemical structure of costunolide.

**Figure 2:**
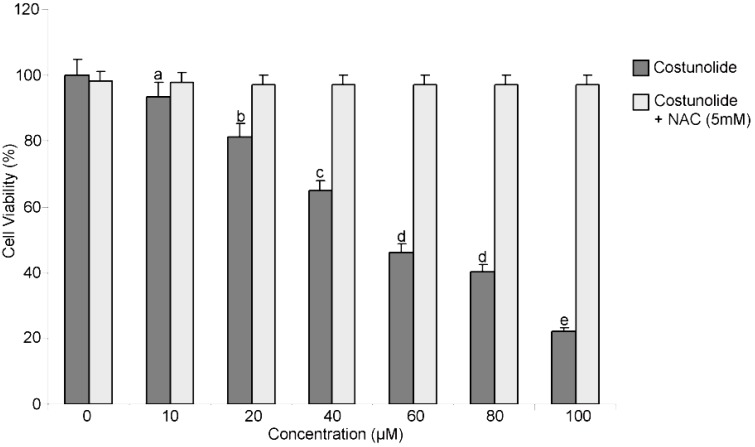
Costunolide inhibited the cell growth and induced cell death. T24 Cells were treated with indicated doses of costunolide in the presence or absence of NAC for 24 h and cell viability was measured by MTT assay. Data are expressed as Mean ± SD (n = 3). Columns not sharing the same superscript letter differ significantly (*p* < 0.05).

To identify a novel and specific inducer of ROS mediated apoptosis in bladder cancer cells, natural compounds were screened in the presence or absence of NAC, a specific ROS scavenger, using the MTT assay. Costunolide, isolated from the roots of *Saussurea lappa* (Mu Xiang), was identified as a potent growth inhibitor of bladder cancer cells. The structure of costunolide is shown in Figure 1. The treatment with costunolide for 24 h inhibited the proliferation of T24 cells in a dose-dependent manner. Pretreatment with 5mM NAC restored the viability of cells indicating that costunolide exerts cytotoxic effect on cell viability through ROS generation (Figure 2).

### 2.2. Costunolide Induced Morphological Changes and Cell Death in T24 Cells

**Figure 3:**
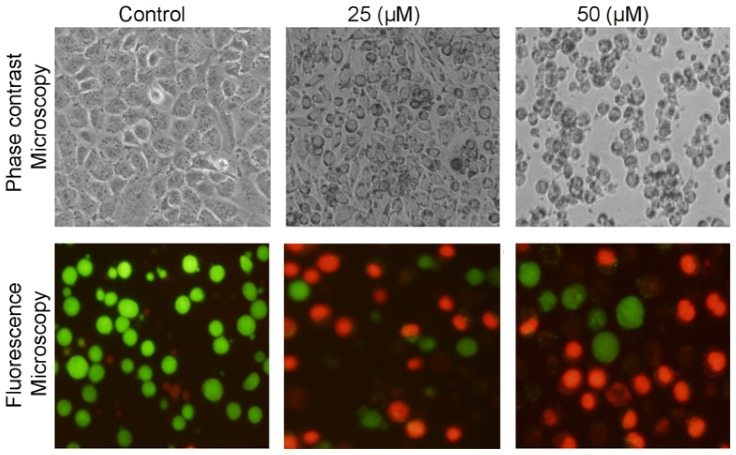
Morphological changes in human bladder cancer T24 cells were observed under phase contrast and fluorescence microscopy after treatment with 0, 25 and 50 μM of costunolide for 24.

Morphological changes were observed under microscopy after treating cells with costunolide resulting in the decreased number of cells as compared to control group and cells became rounded and shrinked, which were polygonal in untreated cells (Figure 3). Furthermore, the antiproliferative effect of costunolide on T24 cells was confirmed by live/dead assay.

**Figure 4:**
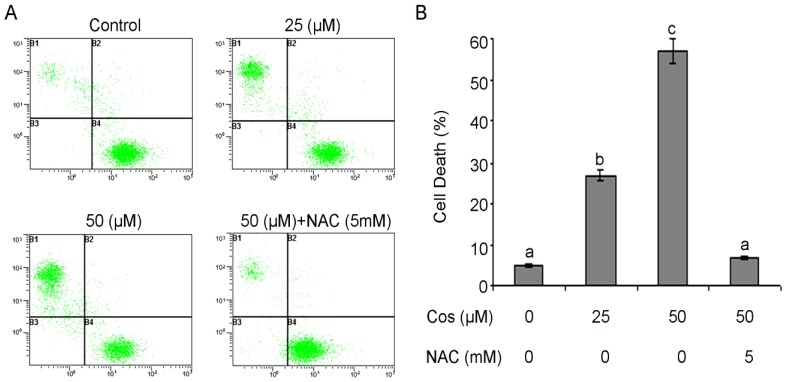
Determination of cell viability by calcein/PI staining and flow cytometry. (**A**) T24 cells were treated with 25 and 50 µM of costunolide in the presence or absence of NAC for 24 h. Histograms show number of PI positive cells (*y*-axis) *vs.* calcein positive cells (*x*-axis). The data shown are representative of three independent experiments with the similar results. (**B**) Data are expressed as Mean ± SD (n = 3). Columns not sharing the same superscript letter differ significantly (*p* < 0.05).

For this purpose, bladder cancer T24 cells were treated with 25 and 50 µM of costunolide for 24 h and live and dead cells were observed using fluorescent probes calcein AM/PI and photographs were taken under fluorescence microscopy (Figure 3). In addition, dead cells were further quantified using fluorescent probes calcein AM/PI and flow cytometry. The results demonstrated that treatment of cells with costunolide decreased the viability of T24 cells in a dose-dependent manner (Figure 4A,B). Costunolide induced growth inhibition of T24 cells in addition to other type of cancer cells previously reported including leukemia [15,19], intestinal carcinoma cells [20], and breast carcinoma cells [21,22].

### 2.3. Costunolide Induced G2/M Cell Cycle Arrest in T24 Cells

Recent insights related to cell cycle regulation indicated that cell cycle progression is tightly controlled by various checkpoints in normal cells while alterations the checkpoints of cell cycle progression lead to aberrant cell proliferation and development of cancer [23].

**Figure 5:**
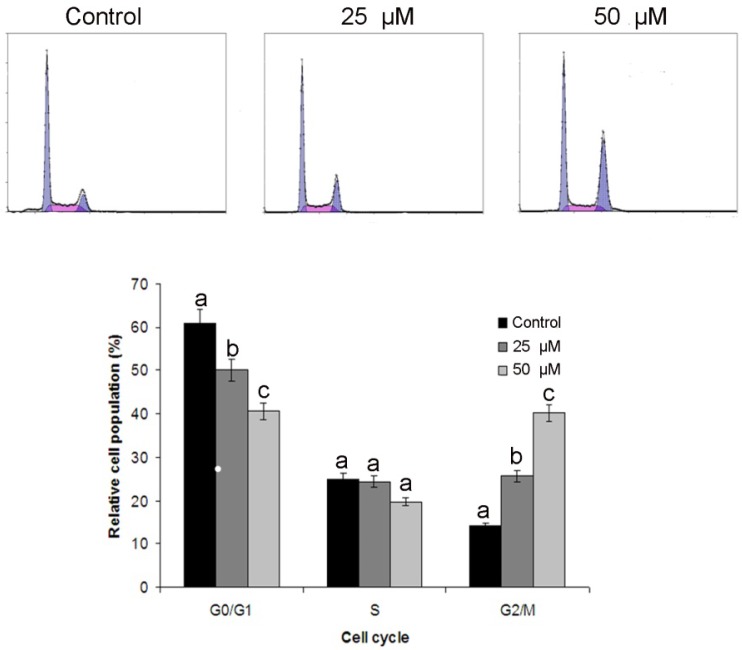
Flow cytometry analysis of cell cycle phase distribution in T24 cells treated with 25 and 50 µM costunolide for 24 h. Data are expressed as Mean ± SD (n = 3). Columns not sharing the same superscript letter differ significantly (*p* < 0.05).

As tumor cells frequently acquire defects in the checkpoints resulting in the deregulation of cell cycle, which lead to unrestrained proliferation. Pharmacological correction of these check points and proper progression of cell cycle is a proficient strategy to control the growth and proliferation of cancer cells [24,25,26]. Next, we analyzed effects of costunolide on cell cycle progression of T24 cells. It was observed that costunolide arrested cell cycle at G2/M phase and the percentage of accumulation of cells in the G2/M phase was increased from 13.78 ± 1.26% in control group to 25.64 ± 2.16% and 41.32 ± 2.66% in the cells treated with 25 and 50 µM of costunolide for 24 h respectively. This increase was coupled with the decreased percentage of cells in G0/G1 phase (Figure 5). These findings are also in line with reported results in other type of cancer cells such as costunolide induced G1-phase cell cycle arrest in human prostate cancer cells [27] and G2/M phase arrest in human hepatocellular carcinoma cells [28].

### 2.4. Costunolide Induced Apoptotic Cell Death in T24 Cells

Apoptosis, autophagy, and necrosis are the major types of cell death [29]. Among the three major pathways of cell death, apoptosis is most well planned and orderly mode of cell death [30,31]. More than 50% of neoplasms undergo aberrations in the apoptotic machinery which leads to abnormal cell proliferation [32,33]. The regulation of apoptosis is, therefore, the most important in the treatment of cancer [34,35,36]. Accumulated evidences indicated that the most of chemotherapeutic agents halt tumor cells proliferation via induction of apoptosis [37,38,39,40].

**Figure 6:**
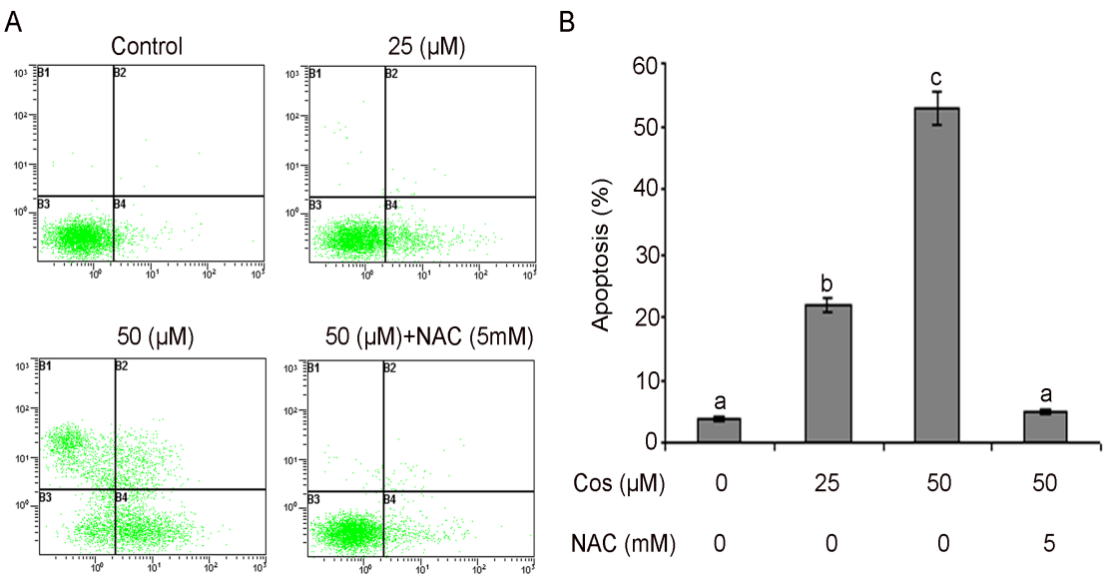
Apoptosis induced by costunolide in T24 cells. (**A**) T24 cells were treated with 25 and 50 µM of costunolide for 24 h in the presence or absence of NAC. Then cells were stained with FITC-conjugated Annexin V and PI for flow cytometric analysis. The flow cytometry profile represents Annexin V-FITC staining in *x* axis and PI in *y* axis. (**B**) Data are expressed as Mean ± SD (n = 3). Columns not sharing the same superscript letter differ significantly (*p* < 0.05).

We examined whether costunolide inhibited cell growth of T24 cells through the induction of apoptosis. Costunolide-induced apoptosis was determined by flow cytometric analysis. Cells were seeded in 12 well plates. After incubation of cells with 25 and 50 µM or without costunolide for 24 h, cells were collected in centrifuge tubes and stained with annexin V-FITC and PI double staining as described in the Experimental section. The results of flow cytometric analysis showed that the rates of apoptosis were 21.43 ± 1.36% and 52.87 ± 1.53% in the cells treated with 25 and 50 µM of costunolide respectively for 24 h as compared to the 4.41 ± 0.42% in control cells. Pretreatment with NAC completely blocked the apoptotic effect of costunolide indicating that induction of apoptosis is a ROS-dependent manner (Figure 6A,B). Costunolide-induced apoptosis in T24 cells was compatible with previously reported studies [8,15,19,41,42].

### 2.5. Costunolide Increased Generation of ROS in T24 Cells

ROS are well known mediators of intracellular signaling of cascades. The excessive generation of ROS can induce oxidative stress, loss of cell functioning, and apoptosis [43]. ROS can also be involved in the process of lipid peroxidation and or the cross linking of thiol groups in proteins; both of these processes can induce the opening of the mitochondrial permeability transition pore (PTP) [44,45]. In the present study, we assumed that costunolide might arouse ROS level, which could be involved in costunolide-induced apoptosis. Therefore, the intracellular ROS level was measured using the ROS-detecting fluorescence dye 2,7-dichlorofluorescein diacetate (DCF-DA) because the DCF assay is highly sensitive, linear, and precise for measuring oxidative stress in irradiated cells [46]. The level of ROS was significantly increased in a dose-dependent manner after treating the cells with costunolide.

**Figure 7:**
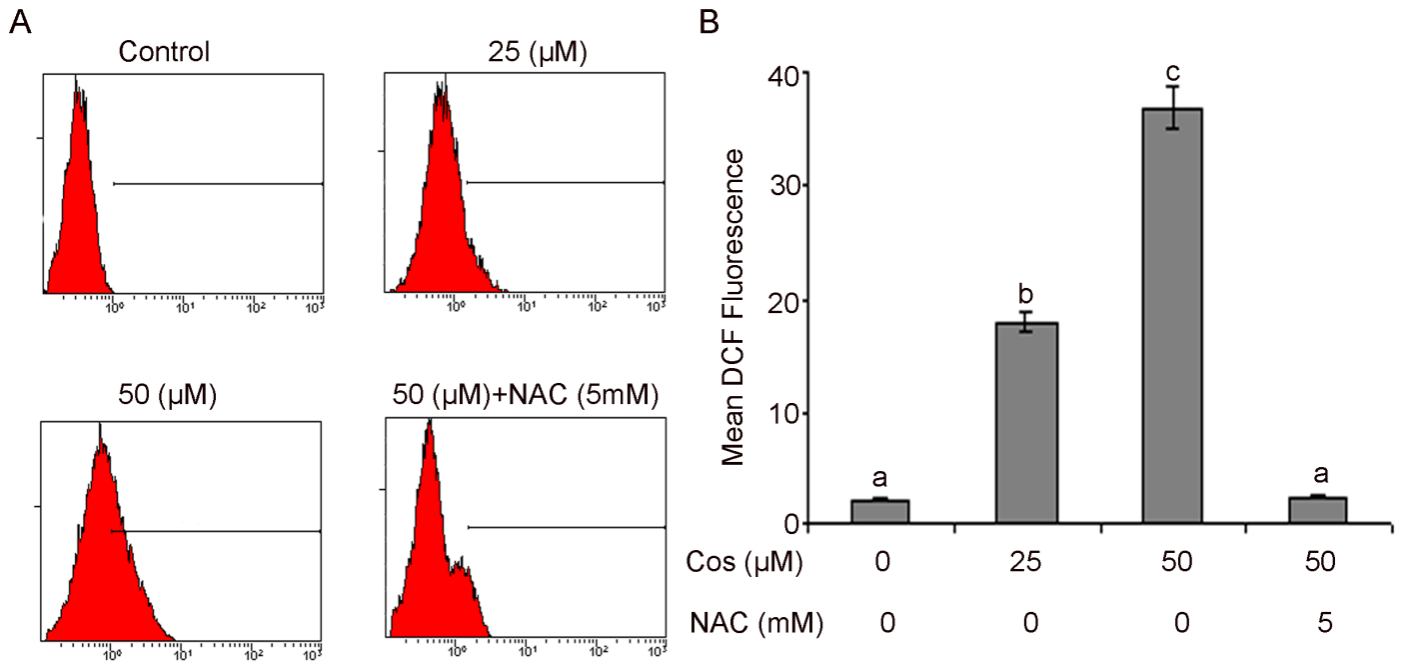
Flow cytometry analysis of ROS generation. (**A**) T24 cells were treated with 25 and 50 µM costunolide in the presence or absence of 5 mM NAC for 24 h. (**B**) Data are expressed as mean ± SD (n = 3). Columns not sharing the same superscript letter differ significantly (*p* < 0.05).

As shown in Figure 7A,B, the ratio of DCF-positive cells, treated with 25 and 50 µM costunolide was significantly higher (18.73 ± 1.65 and 36.80 ± 1.83 *vs*. 1.07 ± 0.53 in control group, *p* < 0.05). The findings evidenced that costunolide had enhanced the generation of ROS in T24 cells. The chemotherapeutic agents causing enhancement in oxidative stress are likely to be toxic to the cancer cells because they are found to be involved in the biological processes like cell cycle arrest, DNA repair, and apoptosis [47].

### 2.6. Costunolide Decreased Mitochondrial Membrane Potential in T24 cells

**Figure 8:**
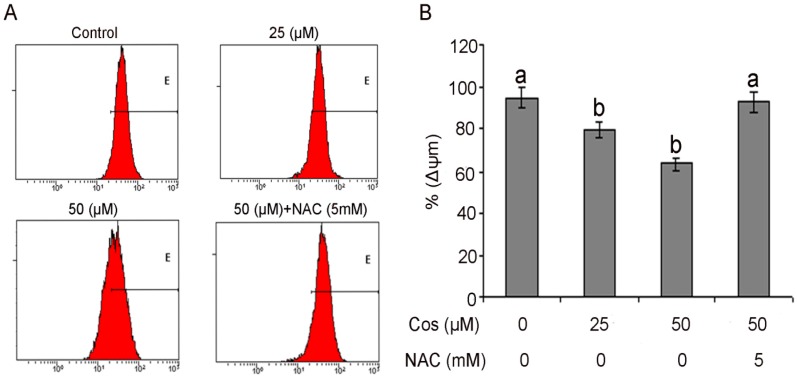
The effects of costunolide on mitochondrial transmembrane potential of T24 cells were determined by flow cytometry. (**A**) The values indicate the percentages of rhodamine 123 fluorescence in the T24 cells treated without and with 25 and 50 µM of costunolide for 24 h in the presence or absence of NAC. (**B**) Data are expressed as Mean ± SD (n = 3). Columns not sharing the same superscript letter differ significantly (*p* < 0.05).

Mitochondria have become an important component of the apoptosis execution machinery, which contain pro-apoptotic proteins (e.g., cytochrome *c*) [30]. It has been elucidated that upon the depolarization of the mitochondrial membrane potential results in the mitochondrial swelling and subsequent release of cytochrome *c* from the intermitochondrial membrane space into the cytosol [48]. It is becoming increasingly apparent that the mitochondria play a fundamental role in the processes leading to cell death [49]. The effects of costunolide on the mitochondrial membrane potential of T24 cells were determined by flow cytometry using rhodamine 123 staining. The rates of depletion of mitochondrial membrane potential were 79.7 ± 1.23% and 66.27 ± 1.42% in the cells treated with 25 and 50 µM of costunolide, respectively, for 24 h as compared to 94.90 ± 0.47% in the control group. To further confirm the involvement of ROS in disruption of mitochondrial membrane potential, cells were treated with 5 mM NAC. Pretreatment with NAC completely prevented dissipation of mitochondrial membrane potential, indicating that this was ROS-dependent (Figure 8A,B).

### 2.7. Costunolide Regulated Apoptosis-Related Proteins in T24 Cells

**Figure 9:**
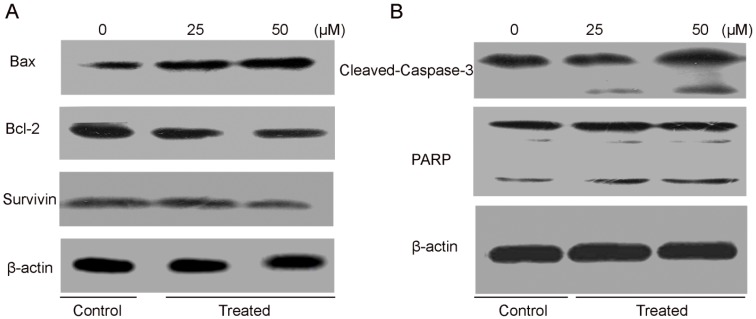
The effect of costunolide on the expression of major apoptosis regulatory proteins. T24 cells were exposed to 25 and 50 µM of costunolide for specified time intervals. Equal amounts of lysate protein were subjected to gel electrophoresis. (**A**,**B**) Expression levels of Bax, Bcl-2, survivin, caspase-3 and PARP were monitored by western blot assay. β-actin was used as loading control. Data are representative of three independent experiments with similar results.

Our data corroborate with the previously reported results that costunolide induced dissipation of mitochondrial membrane potential, which provide the evidence for direct contribution of mitochondria in the costunolide-induced apoptosis [41,42]. Interplay between pro-apoptotic (Bax) and anti-apoptotic (Bcl-2) members of the Bcl-2 family drives the mitochondrial apoptotic pathway [50]. Bcl-2 family proteins are pivotal for increasing the permeability of mitochondrial membranes and the release of cytochrome c, which activates caspases and in turn mobilizes apoptotic cell death [51,52,53]. To investigate the effect of costunolide on expression of Bcl-2, western blotting was done. It was observed that costunolide involved in the down regulation of Bcl-2 in a dose-dependent manner (Figure 9A). These results are similar with previously reported studies [15,41,54]. In addition, we also examined the effect of costunolide on survivin, anti-apoptotic protein. Our results demonstrated that costunolide involved in the down regulation of survivin in a dose-dependent manner (Figure 9A).

The caspases are a family of proteins related to cysteine proteases that are one of the focal executors of the apoptotic process via triggering of the death receptors and mitochondrial pathways to accomplish the programmed cell death [55]. Caspases are present in the form of inactive zymogens those are activated during apoptosis. Among them, caspase-3 is a frequently activated death protease, catalyzing the specific cleavage of many key cellular proteins [56,57]. In order to reveal effects of costunolide on expression of caspase-3 and its downstream target, PARP, western blotting was done. The results showed that procaspase-3 was cleaved to yield 17 and 20 KDa fragments and activation of PARP in treated cells with 25 and 50 µM of costunolide after 24 h as compared to that of control cells (Figure 9B). These findings are supported by previous studies [19,42,54]. These results markedly showed that costunolide induced caspase-dependent cell death in T24 cells.

## 3. Experimental

### 3.1. Chemicals and Reagents

Cell culture medium reagents and MTT [3′-(4,5-dimethyl-thiazol-2-yl)-2,5-diphenyl tetrazolium bromide], propidium iodide (PI), and dimethyl sulfoxide (DMSO) were purchased from Sigma. Fetal bovine serum (FBS) was purchased from the Hangzhou Sijiqing Biological Engineering Materials Co., Ltd. An annexin V-FITC apoptosis detection kit was purchased from Beyotime Institute of Biotechnology (Shanghai, China). Rabbit polyclonal anti-human Bcl-2, Bax, survivin, cleaved caspase-3 and PARP antibodies were purchased from Wuhan Boster Biological Technology Co., Ltd. (Wuhan, China). Mouse anti-β-actin and anti-rabbit antibodies were purchased from Santa Cruz Biotechnology (Santa Cruz, CA, USA). Ponceou and cell lysis buffer for western blots and IP were purchased from Bio SS Beijing (Beijing, China). Rhodamine 123 was purchased from Invitrogen (Eugene, OR, USA).

### 3.2. Extraction, Isolation, and Identification of Costunolide

Costunolide was isolated from the roots of Chinese herb *Saussurea lappa* (Chinese name: Mu Xiang) via fractionation of the extract as we described previously [58,59].

### 3.3. Cell Culture

Human bladder cancer T24 cells were propagated in DMEM nutrients mixture supplemented with 10% FBS and antibiotics at 37 °C in a humidified atmosphere with 5% CO_2_ and 95% air. Cells were seeded in 10 cm culture dish and allowed to grow to approximately 70% confluence before experimentation.

### 3.4. Cell Proliferation Assay

The cytotoxic effects of the costunolide on the cells were determined by the MTT assay. Briefly, T24 cells were seeded at a density of 1 × 10^4^ cells per well in 96-well plates and were allowed to grow overnight. Cells were incubated with 100 µL of complete culture medium containing 10, 20, 40, 60, 80 and 100 µM of costunolide. After incubation for 24 h, growth of cells was determined by adding 10 µL MTT (5 mg/mL in phosphate buffered saline) to each well and incubated for 4 h. After removal of the medium, 150 µL DMSO was added to each well and shaken gently and carefully. The absorbance was read at a wavelength of 490 nm in a plate reader (ELX 800, BIO-TEK Instruments Inc., Winooski, VT, USA).

### 3.5. Morphological Observation under Phase Contrast and Fluorescence Microscope

T24 cells were seeded in 12-well flat bottom microtiter plates and then treated with costunolide at the concentration of 0 25, and 50 µM, respectively. After 24 h of treatment, the morphology of T24 cells was observed under a phase contrast microscope. Furthermore, cells were stained with calcein acetoxymethylester (calcein AM)/PI in the dark for 20 min at room temperature and were observed under fluorescence microscope (Olympus, Tokyo, Japan).

### 3.6. Live/Dead Assay

In order to quantify the live and dead cells, T24 cells were with the fluorescent probes, calcein AM and PI. Calcein AM is cell membrane permeable and stains only viable cells whereas PI is cell membrane impermeable and stains only dead cells. To determine the effect of costunolide, T24 cells were treated with 25 and 50 µM of costunolide in the presence or absence of NAC for 24 h. Subsequently, treated and untreated cells were collected, washed with phosphate buffered saline (PBS) and incubated with PBS solution containing 2 µM calcein AM and 4 µM PI in the dark for 20 min at room temperature. After washing, cells were resuspended in PBS and analyzed for the fluorescence of calcein and PI by flow cytometry (Beckman Coulter, Epics XL, Miami, FL, USA).

### 3.7. Flow Cytometric Analysis of Cell Cycle

For cell cycle analysis, T24 cells were seeded in 12-well plates and then treated with 25 and 50 µM of costunolide for 24 h. After treatments, the percentages of cells in the different phases of cell cycle were evaluated by determining the DNA content after propidium iodide (PI) staining as we described previously [28,60].

### 3.8. Flow Cytometric Determination of Apoptosis

The rate of apoptosis of T24 cells was examined by flow cytometry using annexin V-FITC/PI staining. Briefly, T24 cells were cultured in 6-well plates and allowed to attach overnight. Cells were treated with 25 and 50 µM of costunolide for 24 h. Then cells were collected, washed and resuspended in PBS. Apoptotic cell death was measured by double staining annexin V-FITC and PI using the annexin V-FITC apoptosis detection kit (Beyotime Biotechnology, Shanghai, China) according to the manufacturer’s instructions. Flow cytometric analysis was performed immediately after staining. Data acquisition and analysis were performed by flow cytometry using the Cell Quest software. 

### 3.9. Flow Cytometric Determination of Reactive Oxygen Species (ROS) in T24 Cells

In order to determine the intracellular changes in ROS generation, T24 cells were stained with 2',7'-dichlorofluorescein-diacetate (DCFH-DA). The fluorescent dye DCFH-DA is cell membrane permeable and is converted into the cell membrane impermeable nonfluorescent compound DCFH by intracellular esterases. Oxidation of DCFH by reactive oxygen species produces highly fluorescent DCF. The fluorescence intensity of DCF inside the cells is proportional to the amount of peroxide produced. Briefly, T24 cells were treated with 25 and 50 µM costunolide for 24 h. After treatment, cells were further incubated with 10 μM DCFH-DA at 37 °C for 30 min. Subsequently, cells were harvested, rinsed, re-suspended in PBS, filtered with 300 apertures and analyzed for 2',7'-dichlorofluorescein (DCF) fluorescence by flow cytometry (Beckman Coulter, Epics XL).

### 3.10. Flow Cytometric Determination of Mitochondrial Membrane Potential (Δ*Ψ*m)

To probe the changes in Δ*Ψ*_m_, T24 cells were stained with rhodamine 123 (1 μM) after treatment of 25 and 50 µM of costunolide for 24 h with control group. The fluorescence of rhodamine 123 was measured by flow cytometry with excitation and emission wavelengths of 488 and 530 nm.

### 3.11. Western Blotting

To reveal the mechanism of the apoptotic effect of costunolide, western blotting was done for apoptotic related proteins as previously described [41]. Briefly, T24 cells were incubated with 25 and 50 µM of costunolide for indicated time. Cells were trypsinized, collected in 1.5 mL centrifuge tube and washed with PBS. The cell pellets were resuspended in lysis buffer and were lysed on ice for 30 min. After centrifugation for 15 min, the supernatant fluids were collected and the protein content of the supernatant was measured by the NanoDrop 1000 spectrophotometer (Thermo Scientific, Waltham, MA, USA). The protein lysates were separated by electrophoresis on 12% SDS-polyacrylamide gel and transferred to a PVDF membrane (Amersham Biosciences, Piscataway, NJ, USA). The membranes were soaked in blocking buffer (5% skimmed milk) for 2 h. To probe for BAX, Bcl-2, survivin, cleaved caspase-3, PARP, and β-actin; membranes were incubated overnight at 4 °C with relevant antibodies, followed by appropriate HRP conjugated secondary antibodies and ECL detection.

### 3.12. Statistical Analysis of Data

For the statistical analysis of data, comparisons between results from different groups were analyzed with SPSS for Window Version 15.0. Student’s *t-*test was employed to determine the statistical significance of the difference between different experimental groups and control group. *p* < 0.05 value was defined as statistically significant. All experiments were repeated at least three times. Data were presented as mean ± standard deviation (S.D).

## 4. Conclusions

Although it has been reported that costunolide-induced apoptosis involves the generation of ROS in various cancer cells, our study is the first to describe the role of ROS in the induction of apoptosis in bladder cancer cells. In addition, this study and the pathway that we have described herein is novel and has not been elucidated before. To conclude, costunolide induces cell death in T24 bladder cancer cells via induction of ROS-mediated apoptosis. Analysis of apoptosis-related proteins in T24 cells revealed that costunolide induced the upregulation of Bax and the parallel downregulation of Bcl-2 and survivin expression. This ultimately led to dissipation of mitochondrial membrane potential (ΔΨ_m_) and the sequential activation of caspase-3 and its downstream substrate, PARP, leading to apoptosis. Based on our previous and present studies, we suggest that use of costunolide may represent a new therapeutic strategy for the treatment of human cancers. Further studies are required to support our observations of the anti-tumor potential of this compound, which may represent a promising candidate for *in vivo* studies of mono-therapies as well as combined anti-tumor therapies.
